# Eilat virus displays a narrow mosquito vector range

**DOI:** 10.1186/s13071-014-0595-2

**Published:** 2014-12-17

**Authors:** Farooq Nasar, Andrew D Haddow, Robert B Tesh, Scott C Weaver

**Affiliations:** Institute for Human Infections and Immunity, Center for Tropical Diseases, and Department of Pathology, University of Texas Medical Branch, Galveston, TX 77555 USA; Present Address: Virology Division, United States Army Medical Research Institute of Infectious Diseases, 1425 Porter Street, Frederick, MD 21702 USA

**Keywords:** Alphavirus, Eilat virus, Host range

## Abstract

**Background:**

Most alphaviruses are arthropod-borne and utilize mosquitoes as vectors for transmission to susceptible vertebrate hosts. This ability to infect both mosquitoes and vertebrates is essential for maintenance of most alphaviruses in nature. A recently characterized alphavirus, Eilat virus (EILV), isolated from a pool of *Anopheles coustani* s.I. is unable to replicate in vertebrate cell lines. The EILV host range restriction occurs at both attachment/entry as well as genomic RNA replication levels. Here we investigated the mosquito vector range of EILV in species encompassing three genera that are responsible for maintenance of other alphaviruses in nature.

**Methods:**

Susceptibility studies were performed in four mosquito species: *Aedes albopictus, A. aegypti, Anopheles gambiae,* and *Culex quinquefasciatus* via intrathoracic and oral routes utilizing EILV and EILV expressing red fluorescent protein (−eRFP) clones. EILV-eRFP was injected at 10^7^ PFU/mL to visualize replication in various mosquito organs at 7 days post-infection. Mosquitoes were also injected with EILV at 10^4^-10^1^ PFU/mosquito and virus replication was measured via plaque assays at day 7 post-infection. Lastly, mosquitoes were provided bloodmeals containing EILV-eRFP at doses of 10^9^, 10^7^, 10^5^ PFU/mL, and infection and dissemination rates were determined at 14 days post-infection.

**Results:**

All four species were susceptible via the intrathoracic route; however, replication was 10–100 fold less than typical for most alphaviruses, and infection was limited to midgut-associated muscle tissue and salivary glands. *A. albopictus* was refractory to oral infection, while *A. gambiae* and *C. quinquefasciatus* were susceptible only at 10^9^ PFU/mL dose. In contrast, *A. aegypti* was susceptible at both 10^9^ and 10^7^ PFU/mL doses, with body infection rates of 78% and 63%, and dissemination rates of 26% and 8%, respectively.

**Conclusions:**

The exclusion of vertebrates in its maintenance cycle may have facilitated the adaptation of EILV to a single mosquito host. As a consequence, EILV displays a narrow vector range in mosquito species responsible for the maintenance of other alphaviruses in nature.

**Electronic supplementary material:**

The online version of this article (doi:10.1186/s13071-014-0595-2) contains supplementary material, which is available to authorized users.

## Background

The genus *Alphavirus* in the family *Togaviridae* is comprised mostly of arthropod-borne viruses that utilize mosquitoes as vectors for transmission to diverse vertebrate hosts including equids, birds, amphibians, reptiles, rodents, pigs, humans, and non-human primates [1]. Alphaviruses also have a broad mosquito host range and can infect many species encompassing at least eight genera (*Aedes*, *Culex*, *Anopheles*, *Culiseta*, *Haemagogus*, *Mansonia*, *Verrallina* and *Psorophora* spp.) [2-6]. Recently, a newly characterized alphavirus, Eilat virus (EILV), isolated from a pool of *Anopheles coustani* s.I. mosquitoes was described [7]. EILV is unable to infect and replicate in vertebrate cell lines but can readily replicate in insect cells [7]. The vertebrate host restriction is present at both attachment/entry as well as genomic RNA replication levels [7,8]. EILV is the first “insect-only” alphavirus described and represents a new complex within the genus [7,8].

The lack of vertebrate hosts in its maintenance cycle has likely facilitated EILV adaptation to a single mosquito species; as a consequence EILV may display a narrow vector range. To investigate this hypothesis, we explored the *in vivo* vector host range of EILV by performing susceptibility studies in mosquitoes encompassing three genera that are responsible for maintenance of other alphaviruses in natural transmission cycles: *Aedes albopictus, A. aegypti, Anopheles gambiae,* and *Culex quinquefasciatus.*

## Methods

### Cells and cell culture

C7/10, an *A. albopictus* mosquito cell line, was propagated at 28°C with 5% CO_2_ in Dulbecco’s minimal essential medium (DMEM) containing 10% (v/v) fetal bovine serum (FBS), sodium pyruvate (1 mM), penicillin (100 U/mL), streptomycin (100 μg/mL), and 1% (v/v) tryptose phosphate broth (Sigma).

### cDNA clones and rescue of infectious EILV

EILV and EILV-eRFP cDNA clones were utilized to generate viruses for infection studies. The EILV-eRFP cDNA clone was generated by inserting eRFP under the control of a second subgenomic promoter downstream of the nsP4 gene via SnaB I and SgrA I restriction sites. Viruses were rescued as previously described [8].

### Stability of EILV-eRFP in C7/10 cells

EILV-eRFP was serially passaged in C7/10 cells at a multiplicity of infection (MOI) of 0.1 PFU/cell, in triplicate. After the first passage, virus was titrated and the MOI was adjusted to 0.1 for subsequent passages. Five serial passages were performed, and passages one and five were titrated. Replicates of each passage were also fixed with 2 mL of 2% paraformaldehyde, and plaques expressing eRFP were counted via fluorescent microscopy followed by staining with crystal violet. The percentage of plaques expressing eRFP was calculated [(number of plaques expressing eRFP/total number of plaques) X 100]. Lastly, phase-contrast and fluorescent micrographs were taken of passage one and five virus infection of C7/10 cells.

### Plaque assay

Virus titration was performed on ~80% confluent C7/10 cell monolayers seeded overnight in six-well plates. Duplicate wells were infected with 0.1-mL aliquots from serial 10-fold dilutions in growth medium, 0.4 mL of growth media was added to each well to prevent cell desiccation, and virus was adsorbed for 1 hr. Following incubation, the virus inoculum was removed, and cell monolayers were overlaid with 3 mL of a 1:1 mixture of 2% tragacanth (Sigma) and 2X MEM with 5% FBS (v/v) containing 2% tryptose phosphate broth solution (v/v), penicillin (200 U/mL), and streptomycin (200 μg/mL). Cells were incubated at 28°C with 5% CO_2_ for 3 days for plaque development, the overlay was removed, and monolayers were fixed with 3 mL of 10% formaldehyde in PBS for 30 min. Cells were stained with 2% crystal violet in 30% methanol for 5 min at room temperature (RT); excess stain was removed and plaques were counted.

### One-step replication kinetics

Replication kinetics were assessed in C7/10 cells in triplicate. Infections were performed on 70% confluent monolayers seeded overnight in T-25 cm^2^ flasks. Three replicates of EILV and EILV-eRFP were performed to achieve an MOI of 1 PFU/cell and virus was adsorbed for 2 hr at 28°C. Following incubation, the inoculum was removed, monolayers were rinsed 5 times with RT DMEM to remove unbound virus, and 5 mL of growth medium was added to each flask. Aliquots of 0.5 mL were taken immediately afterward as “time 0” samples and replaced with 0.5 mL of fresh medium. Flasks were subsequently incubated at 28°C and additional time points were taken at 6, 12, 24, and 48 hrs post-infection (hpi). All samples were flash frozen in dry ice/ethanol bath and stored at −80°C.

### Mosquito species

*A. aegypti* (Bangkok, Thailand), *A. albopictus* (Bangkok, Thailand), *C. quinquefasciatus* (Houston, TX, USA), and *A. gambiae* senso stricto (G3 strain) were utilized in these studies. *A. aegypti* and *A. albopictus* were kindly provided by the Armed Forces Research Institute of Medical Sciences (AFRIMS), Bangkok, Thailand.

### Intrathoracic mosquito infections

Cohorts of 15–25 adult females, 5–6 days after emergence from the pupal stage, were cold-anesthetized and inoculated with ~1 μL of EILV at 10^4^-10^1^ PFU/mosquito via the intrathoracic (IT) route. Mosquitoes were given 10% sucrose and held for an extrinsic incubation period of 7 days at 28°C. Whole mosquitoes were placed in 1 mL of DMEM containing 20% FBS (v/v), penicillin (200 U/mL), streptomycin (200 μg/mL), 5 μg/mL amphotericin B, and stored at −80°C. Samples were triturated using a Mixer Mill 300 (Retsch, Newtown, PA), centrifuged at 18,000 × *g* for 5 minutes, and supernatants from each sample were analyzed via plaque assay.

### Imaging mosquito infection

*A. albopictus*, *A. aegypti*, *C. quinquefasciatus,* and *A. gambiae* mosquitoes were injected via IT route with ~1 μL of EILV-eRFP at 10^7^ PFU/mL or with phosphate buffered saline (PBS). Mosquitoes were dissected 7 days post-injection and organs including the anterior and posterior midgut, hindgut, salivary glands, Malpighian tubules, and ovaries were imaged using fluorescent microscopy. PBS-injected mosquitoes were also imaged in the fluorescent field to obtain an exposure time to eliminate background fluorescence. Phase-contrast and fluorescent field photographs were taken of mosquito organs.

### Oral mosquito infections

Cohorts of 100 adult females 5–6 days after emergence from the pupal stage were sugar-starved for 24 hrs [6]. They were fed an artificial meal consisting of defibrinated sheep blood (Colorado Serum Company, Denver, CO) and EILV-eRFP at 10^9^, 10^7^, and 10^5^ PFU/mL [6]. Mosquitoes were allowed to feed for 1 hr, and following feeding mosquitoes were cold-anesthetized and sorted. Fully engorged mosquitoes at or higher than stage 3 were retained for the study [9]. Mosquitoes were given 10% sucrose in cotton balls and held for an extrinsic incubation period of 14 days at 28°C.

### Mosquito processing

Following extrinsic incubation, mosquitoes were cold-anesthetized bodies and legs/wings were removed. Mosquito bodies and legs/wings were triturated separately in 500 μL of 1X DMEM containing 20% FBS (v/v), penicillin (200 U/mL), streptomycin (200 μg/mL), and 5 μg/mL amphotericin B, using a Mixer Mill 300 (Retsch) [10]. Samples were centrifuged at 18,000 × *g* for 5 minutes and supernatants from each sample were analyzed by RT-PCR, eRFP expression, and plaque assays. RT-PCR primers were designed in the nsP4 and capsid genes to flank the eRFP cassette.

## Results

### Plaque size, *in vitro* replication kinetics, and stability of eRFP cassette

**Figure 1:**
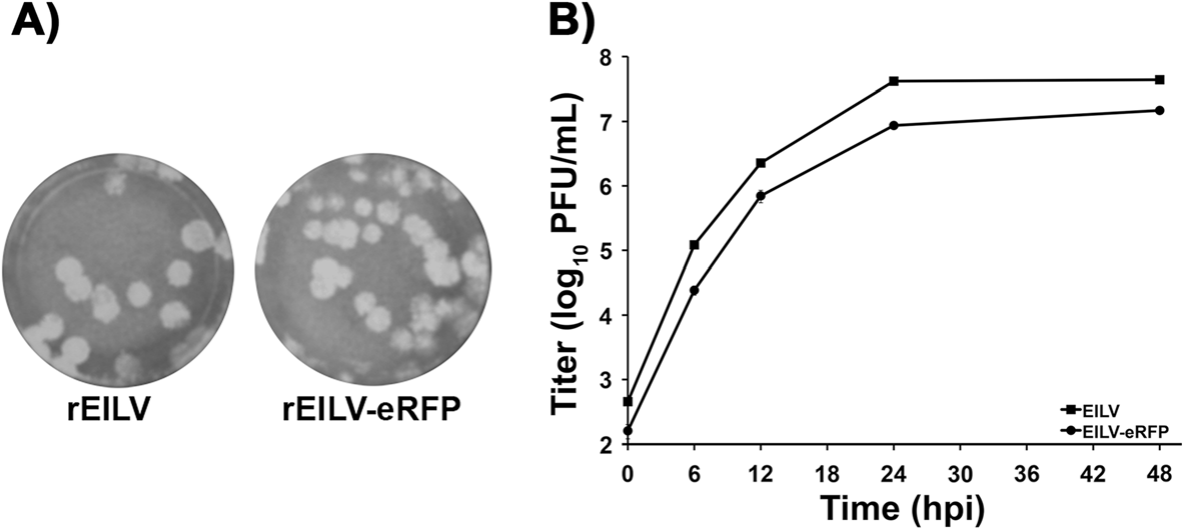
**Comparison of plaque size (A) and replication kinetics (B) of EILV and EILV-eRFP in C7/10 cells (+/−S.D.).**

**Figure 2:**
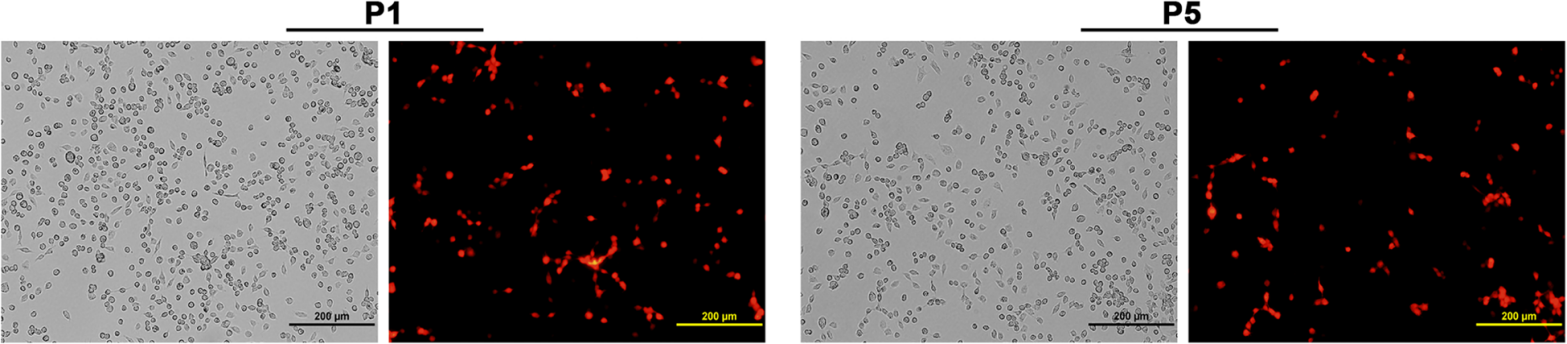
**Stability of eRFP cassette in C7/10 cells after five serial passages.** Phase-contrast and fluorescent photographs of passage one and five infection in C7/10 cells are shown.

EILV and EILV-eRFP assayed on C7/10 cells produced plaques similar in size (~3- to 4-mm) 3 days post-infection (dpi) (Figure 1A). Both EILV and EILV-eRFP displayed similar replication kinetics after infection with an MOI of 1; however, viral titers of EILV eRFP were ~2-8 fold lower than those of EILV (Figure 1B). To investigate the stability of eRFP cassette, EILV-eRFP was serially passaged 5 times in C7/10 cells at an MOI of 0.1. Viral titers at passage one and five were similar (6.1 vs. 6.5 log_10_ PFU/mL), and 99% of plaques expressed eRFP at passage one vs. 90% at passage five (Figure 2 and Table 1).

**Table 1:** **Stability of eRFP cassette in C7/10 cells after five serial passages**

	**Titer**	
**EILV-eRFP**	**(log**_**10**_ **PFU/ml)**	**% of plaques expressing eRFP**
	**(+/− SD)**	
Passage #1	6.1 (+/− 0.18)	99
Passage #5	6.5 (+/− 0.20)	90

Virus titers for passage one and five were generated with standard plaque assay. Percent of plaques expressing eRFP was determined by counting plaques expressing eRFP via fluorescent microscope and crystal violet staining.

### Intrathoracic infection of mosquitoes

**Figure 3:**
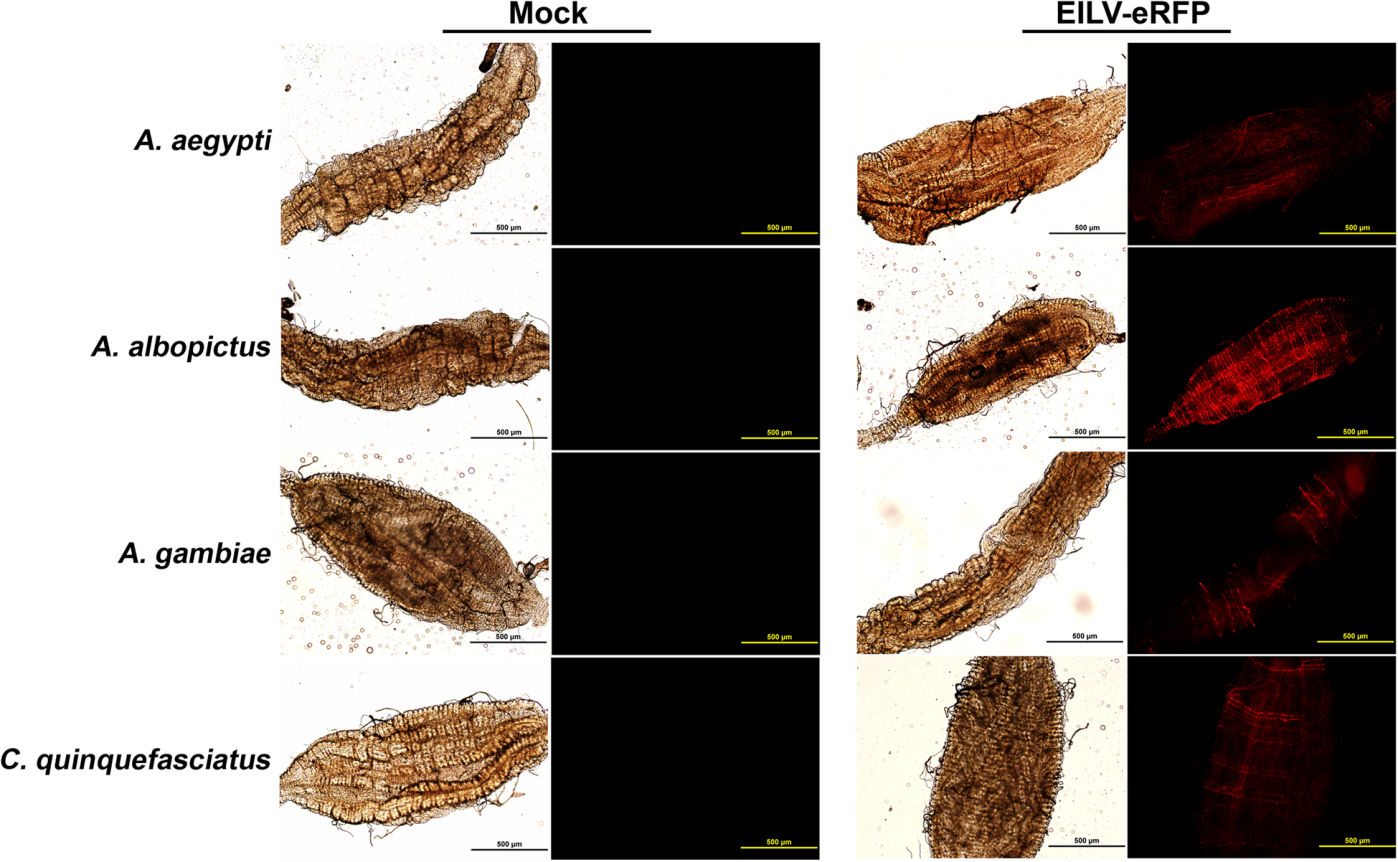
**EILV-eRFP infection of the posterior midgut 7 dpi in mosquitoes infected via IT route at 10** ^**7**^ **PFU/mL.** Phase-contrast and fluorescent photographs were taken at 10X magnification.

**Figure 4:**
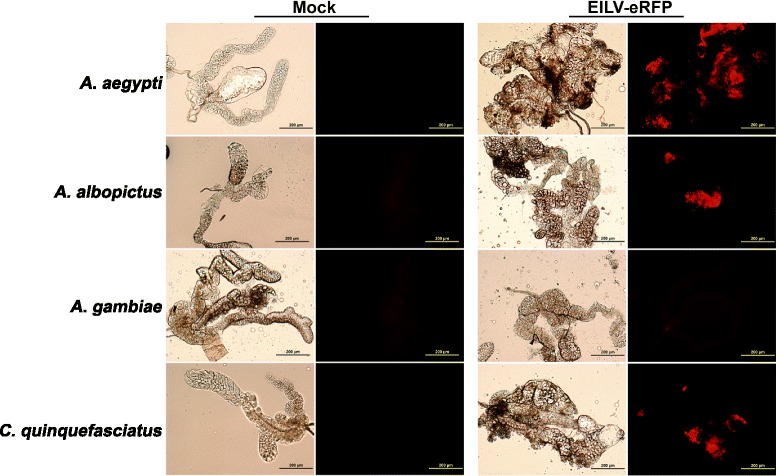
**EILV-eRFP infection of the salivary glands 7 dpi in mosquitoes infected via IT route at 10** ^**7**^ **PFU/mL.** Phase-contrast and fluorescent photographs were taken at 10X magnification.

**Figure 5:**
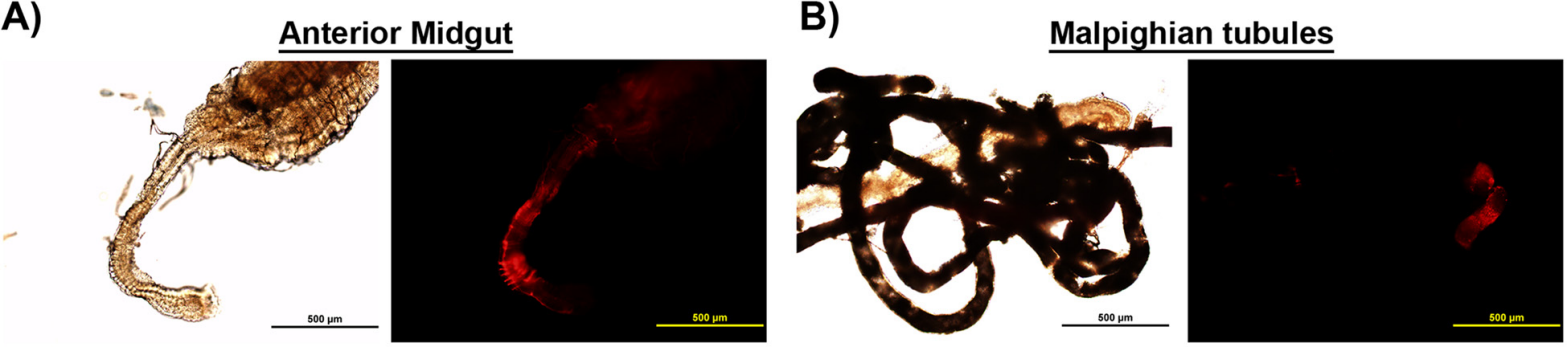
**EILV-eRFP infection of the anterior midgut (A) and Malpighian tubules (B) 7 dpi in** ***A. aegypti*** **mosquitoes infected via IT route at 10** ^**7**^ **PFU/mL.** Phase-contrast and fluorescent photographs were taken at 10X magnification.

To determine the susceptibility of four mosquito species (*A. albopictus, A. aegypti, A. gambiae,* and *C. quinquefasciatus),* they were injected via the IT route with ~1 μL of EILV-eRFP at 10^7^ PFU/mL. Virus replication was detected by visualizing eRFP expression at 7 dpi in various organs including anterior midgut, posterior midgut, hindgut, salivary glands, Malpighian tubules, and ovaries. Organ susceptibility to EILV-eRFP infection varied by species. The posterior midgut was consistently infected in all species at rates of 70-100% (Figure 3, Table 2, Additional file 1: Figure S1). The eRFP expression in the posterior midgut was more pronounced in the midgut-associated muscle tissue (Figure 3, Additional file 1: Figure S1). Salivary glands were the next most susceptible organ, with eRFP expression readily observed in all three *Aedes* and *Culex* species at frequencies of 70-90% (Figure 4, Table 2)*.* Other organs, including the anterior midgut and Malpighian tubules, supported limited or no infection in *A. albopictus and C. quinquefasciatus*, whereas infection rates in both organs ranged from 30-50% in *A. aegypti* (Figure 5, Table 2). In contrast, virus replication was not detected in any organs except the posterior midgut of *A. gambiae* (Table 2). Lastly, virus replication could not be detected in the ovaries of any mosquito species (Table 2).

**Table 2:** **EILV-eRFP infection of various mosquito organs 7 dpi**

**Species**	**Intrathoracic dose**	**Percent infected**					
	**(log**_**10**_ **PFUImL)**	**Anterior midgut**	**Posterior midgut**	**Hindgut**	**Salivary glands**	**Malpighian tubules**	**Ovaries**
*Aedes aegypti*	7.3	50	100	30	90	30	0
*Aedes albopictus*	7.3	30	100	10	90	0	0
*Anopheles gambiae*	7.3	0	70	0	0	0	0
*Culex quinquefasciatus*	7.3	10	90	10	70	0	0

10 mosquitoes/species were visualized with fluorescent microscopy.

**Figure 6:**
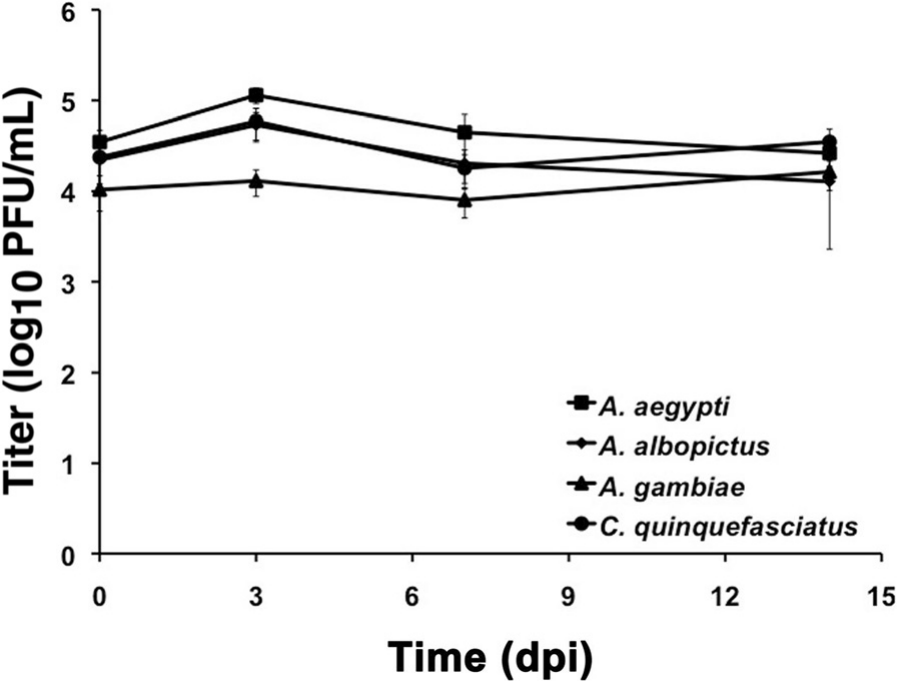
**EILV infection of mosquitoes injected via IT route at 10** ^**4**^ **PFU/mosquito.** N = 5 for each time point.

To determine the mosquito infectious dose 50% (ID_50_) via the IT route, all four species were injected with EILV at 10^4^-10^1^ PFU/mosquito. All species were susceptible at every dose with infection rates of 100% at 7 dpi (Figure 6, Table 3). EILV readily replicated in all four species with a ~1,000-fold increase in virus titers by 7 dpi (Table 3). Thus, similar to other alphaviruses, the ID_50_ of EILV via IT route is <10 PFU/mosquito, indicating a low threshold required to establish infection.

**Table 3:** **Mosquito infection dose 50 (ID**_**50**_ **) via IT route**

**Species**	**Dose**		
	**(log**_**10**_ **PFU/mosquito)**	**Infection (7 dpi)**	
	**(+/− SD)**		**Titer**
		**% infected**	**(log**_**10**_ **PFU/mosquito)**
			**(+/− SD)**
*Aedes aegypti*	4.5 (+/− 0.16)	100	4.6 (+/− 0.15)
	3.7 (+/− 0.11)	100	4.9 (+/− 0.07)
	2.4 (+/− 0.26)	100	4.9 (+/− 0.08)
	1.3 (+/− 0.29)	100	4.8 (+/− 0.15)
*Aedes albopictus*	4.3 (+/− 0.21)	100	4.3 (+/−0.18)
	3.3 (+/− 0.14)	100	4.6 (+/− 0.25)
	2.3 (+/−0.43)	100	4.5 (+/− 0.14)
	1.0*	100	4.4 (+/− 0.19)
*Anopheles gambiae*	4.0 (+/− 0.18)	100	3.9 (+/− 0.43)
	2.8 (+/− 0.18)	100	4.1 (+/− 0.22)
	1.8 (+/− 0.38)	100	4.1 (+/− 0.15)
	1.0*	100	4.3 (+/− 0.04)
*Culex quinquefasciatus*	4.1 (+/− 0.18)	100	4.2 (+/− 0.25)
	3.1 (+/− 0.12)	100	4.2 (+/− 0.24)
	1.8 (+/− 0.30)	100	4.1 (+/− 0.30)
	1.0*	100	4.2 (+/− 0.31)

Mosquitoes were injected at doses ranging from 10^4^-10^1^ PFU/mosquito of EILV. Whole mosquitoes were analyzed post-injection and 7 dpi at each dose via plaques assays. N = 5 for each time point. *Samples were below the limit of detection (10^1^ PFU/mosquito) for the plaque assay.

### Oral infection of mosquitoes

To determine the oral ID_50_, mosquitoes were fed artificial bloodmeals containing EILV-eRFP at 10^9^, 10^7^, and 10^5^ PFU/mL. Virus infection in the bodies and legs/wings was monitored via eRFP expression and plaque assay at 14 dpi. Infection rates in the bodies of *A. albopictus* ranged from 0%-8% at all 3 doses, with average virus titers of 1.5 log_10_ PFU at both 10^9^ and 10^7^ PFU/mL doses (Table 4). Disseminated infection in the legs and wings was not detectable at any dose (Table 4). *A. gambiae* and *C. quinquefasciatus* were susceptible to infection at 10^9^ PFU/mL dose, with body infection rates of 29% and 30%, respectively. Disseminated infection could also be detected in the legs/wings with rates of 21% and 30%, respectively (Table 4). Virus titers were similar in the bodies (1.6 vs. 1.6 log_10_ PFU) as well as legs/wings (1.4 vs. 1.5 log_10_ PFU) in both species (Table 4). At the 10^7^ PFU/mL dose, virus was detected in only 1/23 *A. gambiae* and in none that ingested the 10^5^ PFU/mL dose*.* Virus infection could not be detected in the bodies or legs/wings of *C. quinquefasciatus* at 10^7^ and 10^5^ PFU/mL doses (Table 4). In contrast to other species, *A. aegypti* was susceptible at both 10^9^ and 10^7^ PFU/mL doses, with body infection rates of 78% and 63%, and dissemination rates of 26% and 8%, respectively (Table 4). Similar virus titers were detected at both doses in the bodies (2.3 vs. 2.0 log_10_ PFU) and legs/wings (2.2 vs. 1.5 log_10_ PFU) (Table 4). Virus was not detected in either the bodies or legs/wings at the 10^5^ PFU/mL dose (Table 4).

**Table 4:** **Oral infection of mosquitoes with EILV-eRFP**

**Species**	**Blood meal titer**	**Body**		**Legs/wings**	
	**(log**_**10**_ **PFU/mL)**	**% infected**	**Titer**	**% infected**	**Titer**
			**(log**_**10**_ **PFU)**		**(log**_**10**_ **PFU)**
			**(+/− SD)**		**(+/− SD)**
*Aedes aegypti*	8.9	78 (18/23)	2.3 (+/− 0.87)	26 (6/23)	2.2 (+/− 0.73)
	6.9	63 (15/24)	2.0 (+/− 0.83)	8 (2/24)	1.5 (+/−0.34)
	5.0	0 (0/15)	ND	0 (0/15)	ND
*Aedes albopictus*	8.9	7 (2/27)	1.5(+/− 0.16)	0 (0/27)	1.0*
	6.9	8 (2/24)	1.5(+/− 0.19)	0 (0/24)	1.0*
	5.0	0 (0/31)	ND	0 (0/31)	ND
*Anopheles gambiae*	8.8	29 (4/14)	1.6 (+/− 0.36)	21 (3/14)	1.4 (+/−0.10)
	6.9	4 (1/23)	1.3	4 (1/23)	1.3
	5.7	0 (0/15)	ND	0 (0/15)	ND
*Culex quinquefasciatus*	9.0	30 (9/30)	1.6 (+/− 0.55)	30 (9/30)	1.5 (+/− 0.24)
	7.7	0 (0/28)	1.0*	0 (0/28)	1.0*
	5.8	0 (0/30)	ND	0 (0/30)	ND

Mosquito bodies and legs/wings were analyzed for eRFP expression and plaques assays in C7/10 cells. *Samples were below the limit of detection (10^1^ PFU/mosquito) for the plaque assay.

To further investigate and compare infection rates, mosquito bodies from 10^9^ PFU/mL group were also screened by RT-PCR. Infection rates determined by eRFP expression and plaque assay were identical, whereas the utilization RT-PCR increased rates by ~8%-28% for all species except *C. quinquefasciatus* (Table 5)*.* The utilization of RT-PCR did not significantly change infection rates, however, smaller RT-PCR products could be visualized in some samples indicating loss of the eRFP cassette *in vivo*.

**Table 5:** **Comparison body infection rates via eRFP expression, plaque assay, and RT-PCR**

**Species**	**Oral dose**	**% bodies infected**		
	**(log**_**10**_ **pfu/mL)**	**eRFP**	**Plaque assay**	**RT-PCR**
*Aedes aegypti*	8.9	78 (18/23)	78 (18/23)	87 (20/23)
*Aedes albopictus*	8.9	7 (2/27)	7 (2/27)	15 (4/27)
*Anopheles gambiae*	8.9	29 (4/14)	29 (4/14)	43 (6/14)
*Culex quinquefasciatus*	8.9	30 (9/30)	30 (9/30)	30 (9/30)

Percent of mosquito bodies positive at 14 dpi.

## Discussion

Alphaviruses expressing fluorescent proteins are powerful tools for the study of mosquito infection [11-17]. The fluorescent protein cassettes expressed by engineered viruses [Sindbis (SINV) and chikungunya (CHIKV)] are stable both *in vitro* and *in vivo* [11,12]. These viruses display similar replication kinetics, infection and dissemination rates in mosquitoes as wild-type (wt) viruses without expression cassettes [11,12]. Consequently, they have been utilized to study infection dynamics, susceptibility of various mosquito organs, and potential bottlenecks during virus spread *in vivo* [11-17]. To perform similar studies, a EILV clone was engineered to express eRFP via an additional subgenomic promoter downstream of nsP4 gene, a genetic placement that has been shown to be stable both *in vitro* and *in vivo* for SINV and CHIKV [11,12]. EILV-eRFP displayed similar plaque size and replication kinetics relative to wt EILV in C7/10 cells. The eRFP cassette was shown to be stable *in vitro* over 5 serial passages, where 90% of the plaque population retained eRFP expression, a stability level comparable to previous results with SINV and CHIKV [11,12]. Virus replication could be visualized in the anterior and posterior midgut, hindgut, salivary glands, and Malpighian tubules. EILV-eRFP could also be recovered from bodies and legs/wings collected 14 days after oral infection. Infection rates determined by eRFP expression and plaque assay were identical, indicating that the eRFP expression cassette was stable *in vivo*. Although the proportion of the virus population expressing eRFP *in vivo* at 14 dpi was not determined, the detection of smaller RT-PCR products suggested that a portion of the virus population lost eRFP expression as observed in other studies [12,13].

The aim of our work was to investigate infection of an insect-only alphavirus, EILV, in mosquito species encompassing three genera that are responsible for maintenance of alphaviruses in nature. Most mosquito species are susceptible to mosquito-borne alphavirus infection via the IT route; however, their oral susceptibility can vary greatly even within a species collected from different geographical locations [2,18-28]. Principal enzootic vectors utilized for transmission can often be infected orally with only a few infectious particles, while other species may require 100–1,000-fold higher dose to establish infection, or may be completely refractory to infection [18,25-28]. Our results were consistent with these findings, as all four species were susceptible to infection via the IT route. EILV infected several mosquito organs, with the posterior midgut and salivary glands consistently infected via the IT route in most species. Infection in the posterior midgut after IT inoculation was mainly associated with longitudinal and circular muscles, where the eRFP signal was more pronounced. The anterior midgut, hindgut, Malpighian tubules, and ovaries were either refractory to infection or supported minimal replication. *A. aegypti, A. gambiae,* and *C. quinquefasciatus* required high oral infectious dose (10^9^ PFU/mL), and *A. albopictus* was almost completely refractory to oral infection. Lastly, EILV was able to disseminate from the midgut following oral exposure, albeit only after a large oral infectious dose (10^9^ PFU/mL).

Mosquito-borne alphaviruses are maintained in nature in enzootic or endemic cycles between susceptible mosquito vectors and vertebrate hosts. In this two-host cycle, adaptive virus evolution may be constrained by trade-offs required for efficient infection of widely divergent hosts [29-34]. However, EILV is restricted at both attachment/entry and RNA replication stages of infection in vertebrate cells, and is therefore unlikely to be maintained in a two-host cycle [7,8]. The lack of vertebrates in its maintenance likely removed the evolutionary “trade-off” pressures that typically constrain two-host alphaviruses from optimal adaptation to a single host. Assuming that vertical transmission is its main mode of maintenance in nature, EILV may have adapted to a single mosquito host, resulting in a narrow vector range. This species-specific adaptation and lack of a need for oral infection in nature could eliminate or greatly reduce EILV oral susceptibility even at high doses (10^9^ PFU/mL) resulting in limited tissue susceptibility required of other arboviruses that must disseminate and infect the salivary glands for transmission. Our data support this hypothesis. EILV titers after IT infection were ~10-100 fold less than reported for other alphaviruses, and infection was limited mainly to midgut-associated muscle tissue and salivary glands [35-37]. Most of the mosquito species were either refractory or supported limited infection via the oral route even at 10^9^ PFU/mL. Additionally, infection in the most susceptible species, *A. aegypti*, was limited to the midgut at both 10^9^ and 10^7^ PFU/mL doses, suggesting that EILV was unable to disseminate into the hemocoel. However, this hypothesis needs to be investigated further through field collections to determine the EILV host range in nature.

Natural maintenance of EILV presumably relies on vertical and possibly venereal transmission. These mechanisms are reported to occur for other alphaviruses; however, estimated rates are low and are consequently thought to play a minor role in natural maintenance [38-43]. Similarly, vertical and venereal transmission have been demonstrated for mosquito-only flaviviruses [44-46]. However, in contrast to alphaviruses, the vertical transmission rate of Culex flavivirus in naturally infected mosquitoes has been shown to be almost 100% and is consequently thought to play a major role in natural maintenance [45]. A critical step in the vertical transmission of insect-only viruses would involve infection of the ovaries. Accordingly, studies with Culex flavivirus have detected viral RNA in the ovaries of F1 progeny of infected females and intrathoracically infected mosquitoes [45]. However, we were unable to detect EILV infection in the ovaries of four mosquito species tested via the intrathoracic route. These data further support the narrow vector range hypothesis. The identification of principal vector species will enable studies to determine the mechanism/s of maintenance in nature.

EILV was isolated from a pool of *A. coustani* s.I. in a survey of the Negev desert in Israel during 1982–1984 [47]. Its apparent lack of a vertebrate host suggests that *A. coustani* s.I. may be the only mosquito species responsible for natural maintenance of EILV. If true, EILV would be only the second alphavirus described that utilizes *Anopheles spp.* as a natural vector. The other is o’nyong-nyong virus (ONNV), an African alphavirus transmitted to humans by *A. gambiae* and *A. funestus*, which has caused large epidemics of severe arthralgia [48-51]. *A. coustani* s.I. is found in Africa and parts of the Middle East; however, it is not a well-studied species [52]. Limited data indicate that it can exhibit both zoophilic and anthropophilic feeding behavior [53,54]. It is also a secondary vector for malaria parasite transmission in Africa but its role in transmission of other human and/or animal pathogens remains poorly understood [55-58]. Whether *A. coustani* s.I. is the principal species responsible for maintenance of EILV in nature needs to be investigated further by virus isolation from field collected mosquito, larvae and eggs. It is possible that *A. coustani* s.I. is not the principal vector, and other mosquito species near the sites of the original survey study need to be sampled in order to identify the principal vector [47].

## Conclusions

The present study shows that the exclusion of vertebrates in its maintenance cycle likely facilitated the adaptation of EILV to a single mosquito host. As a consequence, EILV displays a narrow vector range in mosquito species responsible for the maintenance of other alphaviruses in nature.

## Additional file

Additional file 1: Figure S1. EILV-eRFP infection of the posterior midgut 7 dpi in mosquitoes infected via IT route at 10^7^ PFU/mL. Phase-contrast and fluorescent photographs were taken at 40X magnification.
