# Supplementary figures and images for: Eilat virus displays a narrow mosquito vector range
Source: Parasit Vectors. 2014 Dec 17;7:595. doi: 10.1186/s13071-014-0595-2 (PMC4297418; doi:10.1186/s13071-014-0595-2)

***A. aegypti***

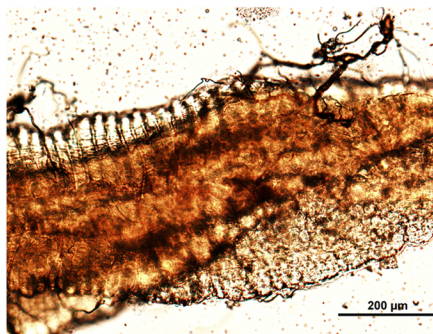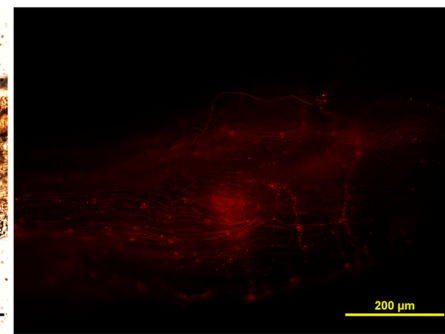

***A. albopictus***

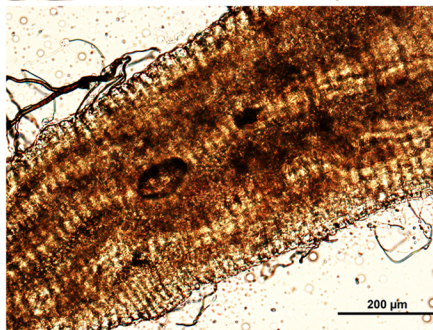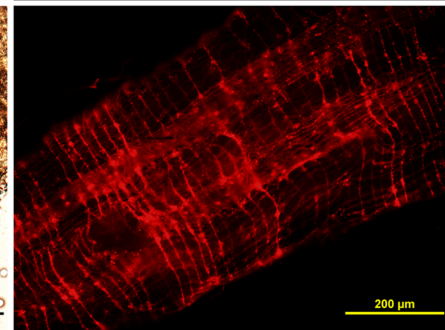

***A. gambiae***

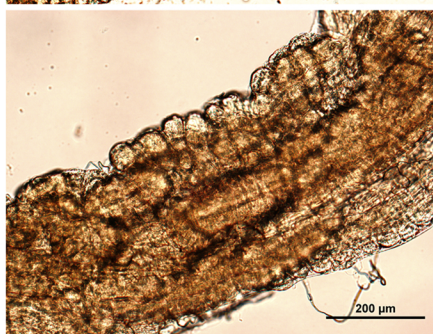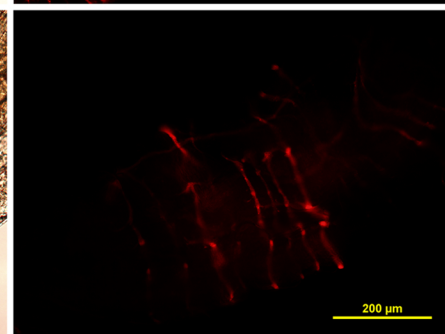

***C. quinquefasciatus***

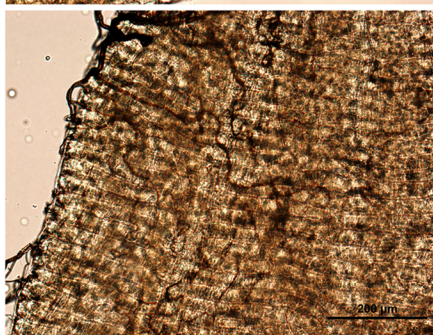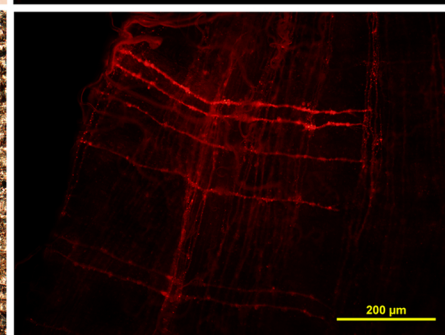

Supplement: Additional file 1: Figure S1. — EILV-eRFP infection of the posterior midgut 7 dpi in mosquitoes infected via IT route at 107 PFU/mL. Phase-contrast and fluorescent photographs were taken at 40X magnification. [file 13071_2014_595_MOESM1_ESM.pdf]
